# Biomimetic device and foreign body reaction cooperate for efficient tumour cell capture in murine advanced ovarian cancer

**DOI:** 10.1242/dmm.043653

**Published:** 2020-06-17

**Authors:** Lorena Alonso-Alconada, Alexandre de la Fuente, María Santacana, Alba Ferreiros, Rafael Lopez-Lopez, Xavier Matias-Guiu, Miguel Abal

**Affiliations:** 1 Translational Medical Oncology (oncomet), CIBERONC, Health Research Institute of Santiago (IDIS), University Hospital of Santiago (SERGAS), Santiago de Compostela 15706, Spain; 2 Department of Pathology and Molecular Genetics, Hospital Universitari Arnau de Vilanova, University of Lleida, IRBLleida, CIBERONC, Lleida 08080, Spain

**Keywords:** Biomimetics, Foreign body reaction, Ovarian cancer, Peritoneal metastasis, Tumour cell capture

## Abstract

Metastasis is facilitated by the formation of pre-metastatic niches through the remodelling of the extracellular matrix (ECM) promoted by haematopoietic and stromal cells. The impact of these primed sites is pronounced for intraperitoneal metastases, where the cavity-exposed ECM supports the attachment of the disseminating tumour cells. Likewise, implantation of biomaterial scaffolds influences metastatic progression systemically through a foreign body reaction (FBR). In this study, we integrated the concept of creating an artificial niche to capture tumour cells actively disseminating in the peritoneal cavity with a therapeutic strategy modulating the interactions of metastatic cells with the ECM. The aim was to transform a disseminated disease into a focal disease. For this, we designed and developed a ‘biomimetic’ ECM composed of a nonresorbable three-dimensional scaffold with collagen coating and characterized the FBR to the implanted biomaterial. We also analysed the safety of the implanted devices and their ability to capture tumour cells in different murine preclinical models of advanced ovarian cancer. Implantation of the biomimetic devices resulted in an initial inflammatory reaction that transformed progressively into a fibrous connective tissue response. The adhesive capabilities of the scaffold were improved with the ancillary effect of the FBR and showed clinical utility in terms of the efficacy of capture of tumour cells, disease focalization and survival benefit. These results demonstrated the performance and safety of this ‘biomimetic’ ECM in preclinical models of advanced ovarian cancer. Translated into the clinical setting, this new therapeutic strategy represents the possibility for control of peritoneal carcinomatosis upon primary ovarian debulking surgery and to expand the percentage of patients who are candidates for second rescue surgeries at the time of relapse.

## Abstract (precis)

**Editor's choice:** A new therapeutic strategy targeting peritoneal metastasis based on biomimetic devices that capture disseminating tumour cells is used as a proof of concept in clinically relevant models of advanced ovarian cancer.

## INTRODUCTION

The concept of ‘pre-metastatic niches’ refers to the conditioning of future sites of metastasis or ‘soil’ in preparation for the reception of tumour cells, and the crosstalk of tumours with the environment at distant sites to improve the efficiency of the process of metastasis (Peinado et al., 2017; Mikuła-Pietrasik et al., 2018). A combination of hypoxia-derived factors, mobilization of precursor cells and remodelling of the target tissue results in a specialized microenvironment that facilitates and promotes the invasion, survival and outgrowth of disseminated tumour cells (Sleeman, 2012). The impact of these primed sites for the implantation of metastatic cells is particularly pronounced for intraperitoneal metastases. Patients presenting with tumour cell dissemination on the peritoneal surfaces of the abdomen, such as gastrointestinal and gynaecological malignancies, face drastically worse prognosis (Colombo et al., 2006; Lemoine et al., 2016; Lambert, 2015).

Among gynaecological malignancies, ovarian cancer is usually diagnosed at an advanced stage, when tumours have spread in diffuse peritoneal lesions that impede surgical removal. Nowadays, the survival rate at 5 years in advanced ovarian cancer does not exceed 25% (Siegel et al., 2018). The biology of ovarian cancer is different from that of most other solid tumours, predominantly confined within the abdominal cavity (Lengyel, 2010). Generally, ovarian cancer is only superficially invasive, although advanced disease is characterized by large intra-abdominal tumours in the ovary and the omentum. The primary mechanism for peritoneal dissemination involves cancer cells detaching from the primary site and the peritoneal fluid carrying these cells to secondary sites of implantation, with the omentum as the most common site of metastasis. In advanced ovarian cancer, recurrences are often located within the abdominal cavity, suggesting the role of a residual niche within the peritoneum. This peritoneal niche also attracts circulating tumour cells preferentially implanting and metastasizing in the omentum (Pradeep et al., 2014). The creation of peritoneal niches involves impairment of the integrity of the single layer of mesothelial cells covering an underlying stroma composed of extracellular matrix (ECM) and stromal cells (Kenny et al., 2014). Exposure of ECM to tumour cells disseminating through the peritoneal cavity promotes their attachment and homing, with collagen I as a major constituent of the submesothelial ECM and the preferred substrate for cancer cell attachment (Sodek et al., 2012; Lu et al., 2012; Worzfeld et al., 2017).

A new cancer cell capture technology was recently developed to redirect peritoneal metastasis by offering an optimal microenvironment that competes with the natural niches of tumour cell homing. The initial design combined an implantable scaffold coated with exosomes as the capture agent, resulting in tumour cell attachment as its nonpharmacological mode of action (de la Fuente et al., 2015). Extracellular vesicles, including exosomes, participate in cellular communication and, among other roles, in pre-metastatic niche formation (Peinado et al., 2011). In addition, it is known that the implantation of a biomaterial scaffold generates a foreign body reaction (FBR) that includes inflammation and wound-healing responses (Anderson et al., 2008). The FBR has a systemic impact on immune cell phenotype, mainly focused on the innate immune cells although the adaptive immune response may also be influenced, and alters components of the tumour stroma, including the cancer-associated fibroblast phenotype and ECM composition (Aguado et al., 2018). Based on these premises, we have now integrated the concept of creating an artificial niche, which might remodel the pattern of tumour dissemination and transform a disseminated disease into a localized disease, with therapeutic strategies targeting the tumour microenvironment to modulate cancer cell interactions with the ECM and mesothelial cell retraction (Hansen et al., 2016; Høye and Erler, 2016). For this, we explored the impact of the FBR generated by a ‘biomimetic’ ECM technology, characterizing this artificial tumour microenvironment as a modulator of cancer cell interactions that serves as a preferential niche for tumour cell homing.

## RESULTS

### Mode of action: a synthetic and durable metastatic niche

**Fig. 1.**
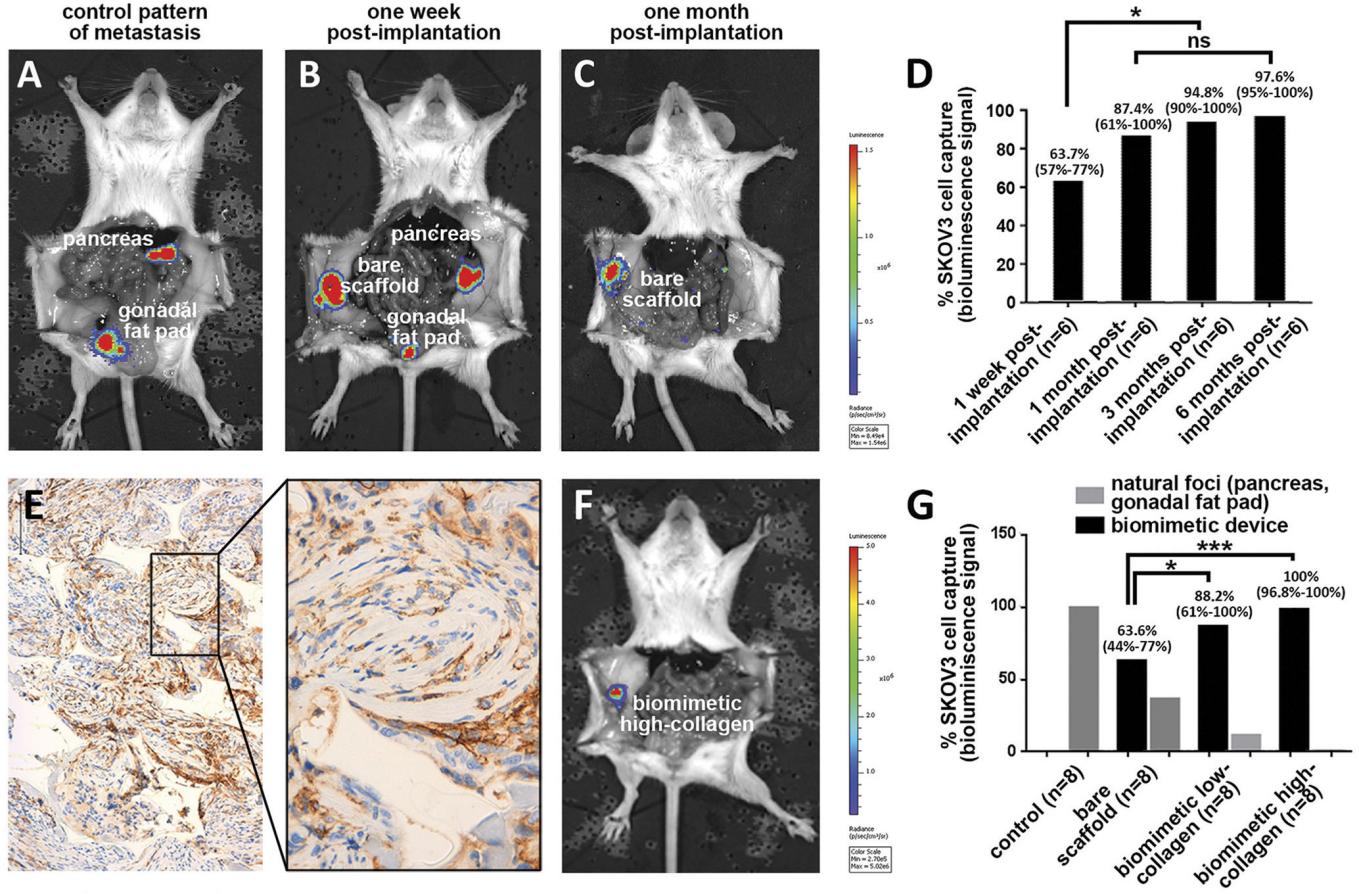
**Contribution of components of the biomimetic device to the capture efficacy of the device in the mouse model of ovarian peritoneal dissemination.** (A-C) Representative images showing the natural pattern of peritoneal metastasis (A) 1 week after intraperitoneal injection of luciferase-expressing SKOV3 cells, compared with the focalization of the disease within the bare scaffold implanted (B) 1 week and (C) 1 month before tumour cell injection; normalized photons represented in the colour scale are indicative of the corresponding tumour cells for each animal included in the three study groups. (D) Quantification of the efficacy of tumour cell capture of the bare scaffold represented as a percentage of SKOV3 cells within the scaffold implanted 1 week and 1, 3 and 6 months before tumour cell injection (*n*=6; data are expressed as the median percentage±range; *P*=0.002). (E) LYVE1 immunohistochemistry of the bare scaffold, showing infiltration of fibroblasts, owing to the FBR, into the implanted device at 3 months post-implantation (scale bar: 500 µm). Right image is a ×40 magnification of the indicated area. (F) The contribution of the collagen coating to the efficacy of tumour cell capture of the bare scaffold is shown in the representative bioluminescence image of complete focalization of peritoneal metastasis within the biomimetic device; colour scale is indicative of the corresponding tumour cells. (G) Histogram represents the quantification of the bioluminescence signal from tumour cells captured by the biomimetic device as a function of the device components (bare scaffold plus increasing concentrations of the collagen coating) and the natural sites of metastasis (pancreas and gonadal fat pad) for the mice (*n*=8) included in each group of the study (expressed as the median percentage±range). ns, not significant; *P<0.05, ***P<0.001.

The impact of the known FBR described in association with porous synthetic biomaterials on the efficacy of tumour cell capture of bare nonresorbable scaffolds was studied in a murine model of ovarian cancer peritoneal dissemination. The scaffolds were surgically implanted at the inner wall of the peritoneum, and the capture efficacy of 1 million intraperitoneally injected luciferase-SKOV3 ovarian cancer cells was evaluated with time. In the absence of any device, sacrificed mice presented a pattern of peritoneal metastasis with the fat surrounding the diffuse pancreas and the uterine fat as the natural sites for luciferase-SKOV3 cell implantation (Fig. 1A). The implantation of the bare scaffolds in the peritoneum opposite to these natural sites of metastasis 1 week before SKOV3 cell injection resulted in a reduced metastasis pattern and focalization of peritoneal disease within the scaffold (Fig. 1B). More interestingly, the capture efficacy of the bare scaffolds increased with time, showing a complete remodelled pattern after long-term implantation (Fig. 1C). The bioluminescence signal at the devices increased from 63.7% of captured tumour cells at 1 week post-implantation (*n*=6; Fig. 1D) to 87.4% at 1 month (*n*=6; *P*=0.002; Fig. 1D) and 94.8 and 97.6% capture efficacy at 3 and 6 months post-implantation, respectively (*n*=6; Fig. 1D). No side effect or adverse effect of the implanted device on the animals was observed, evaluated according to the guidelines of the Directive 2010/63/EU on the protection of animals used for scientific purposes, including assessment of body weight, coat condition, breathing, environment (stools), behaviour and locomotion. Of note, immunohistochemistry showed that this improved capture efficacy at the 3-month post-implantation time point was correlated with the recruitment of fibroblasts into the bare scaffolds and their differentiation into myofibroblasts, as evidence by lymphatic vessel endothelial hyaluronan receptor 1 (LYVE1) staining (Fig. 1E).

Given that this FBR characterized by fibroblast-to-myofibroblast transition is associated with matrix deposition and elevated collagen synthetic capacity that results in fibrosis, we speculated that in addition to the reticulated structure of the scaffolds favouring the *in vivo* homing of tumour cells, the production of ECM proteins would improve the capture efficacy of the bare scaffolds. We thus designed a biomimetic device composed of a nonresorbable scaffold with a type I collagen coating (Fig. S1). The additive contribution of each of the two elements of the biomimetic device (scaffold+coating) was confirmed both *in vitro* in an orbital adhesion assay, which mimics peritoneal dissemination in ovarian cancer (Fig. S2), and *in vivo*, reproducing the complete remodelling of the pattern of peritoneal dissemination. The peritoneal metastases were localized in a unique location within the biomimetic device 1 week after implantation (Fig. 1F). Quantification of the bioluminescence signal confirmed an increased efficacy of tumour cell capture of the bare scaffolds as the collagen coating concentration was increased (Fig. 1G). In the biomimetic low-collagen group, devices manufactured with a low concentration of collagen (25 mg) captured 88.2% of the injected cells (Fig. 1G; *n*=8), an improved adhesive capacity compared with 63.6% for the bare scaffold (*P*=0.033; Fig. 1G; *n*=8). Biomimetic devices from the high-collagen group with the targeted collagen coating level designed for clinical use (250 mg) captured 100% of SKOV3 tumour cells injected (*P*=0.004; Fig. 1G; *n*=8). We have also demonstrated the efficacy of the biomimetic technology to capture other clinically relevant tumour cells disseminating in the peritoneal cavity, independent of their tumour origin. Tumour cells lines representative of carcinoma serous and endometrioid histology, in addition to patient-derived ovarian tumour cells, were effectively captured by the biomimetic devices with the targeted collagen coating level designed for clinical use, resulting in the transformation of a peritoneally disseminated disease into a focal disease (Fig. S3). Together, these findings support the adhesive, nonpharmacological mode of action of the device, with the principal tumour cell capture action provided by the bare scaffold and an ancillary improved capture efficacy provided by the collagen coating, overall recapitulating a metastatic niche.

**Fig. 2.**
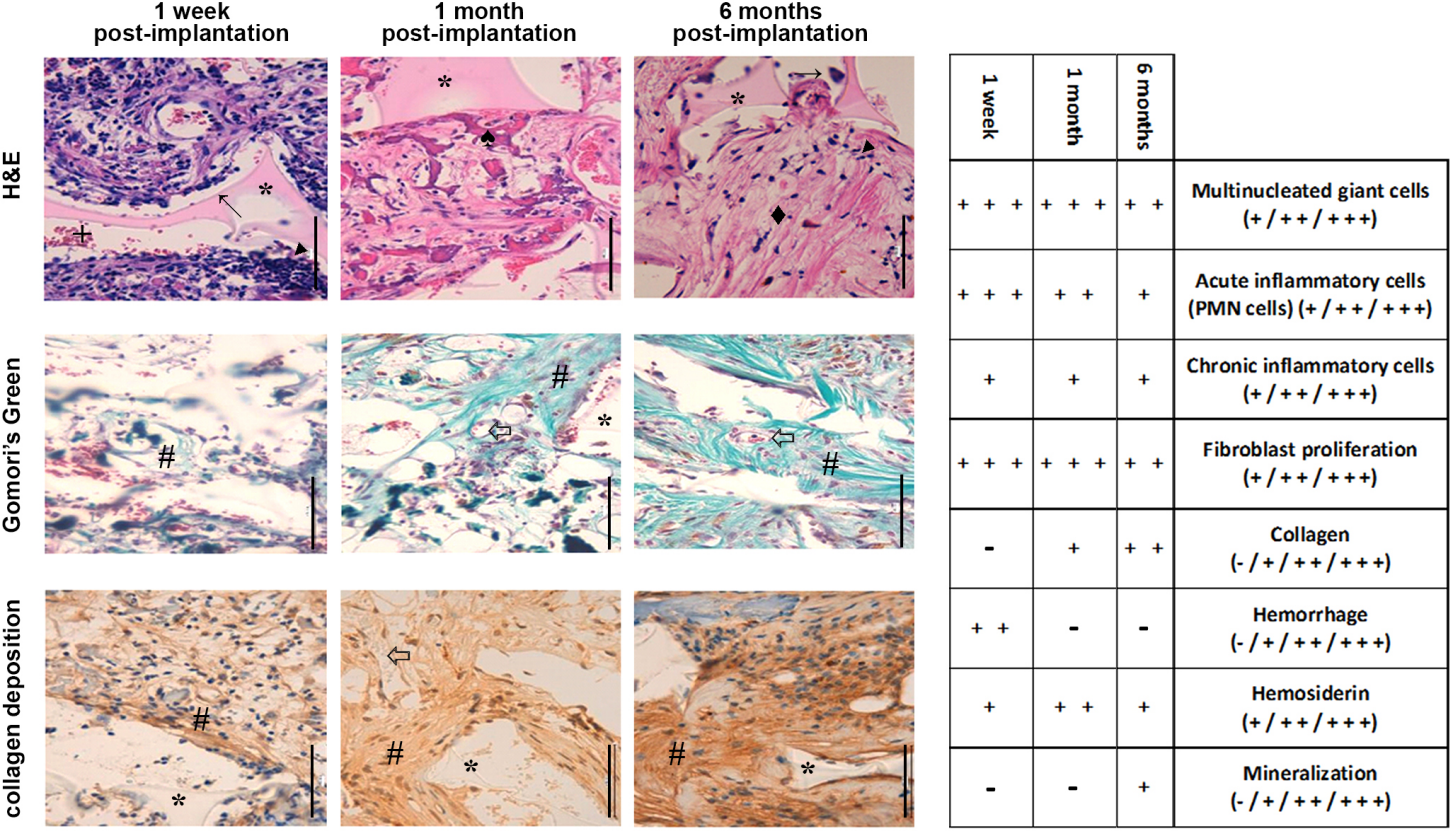
**Characterization of the foreign body reaction associated with the implantation of the biomimetic devices in the peritoneal cavity of mice.** (Left panels) Representative images of the FBR to the devices 1 week after implantation, showing a dense cellular infiltration into the inner layer of the device directly facing the peritoneal wall. Histological (H&E) staining (nuclei, blue/purple; cytoplasm, pink; collagen, pale pink) showed an initial inflammatory response followed by a fibrotic response directly associated with the device (scale bars: 500 µm). Symbols are as follows: biomimetic device (*), multinucleated giant cells (**→**), infiltrating polymorphonuclear (PMN) cells (◄), haemorrhage (+), collagen deposition (♦), haemosiderin (♠), fibrous tissue formation (**#**) and blood vessels (⇦). (Right summary table) H&E staining indicated a moderate to marked acute inflammatory response at 1 week post-implantation, with abundant multinucleated giant cells. The amount of inflammatory cells (acute inflammatory cells, chronic inflammatory cells and multinucleated macrophages), the presence of erythrocytes, haemosiderin deposition, mineralized deposits and the amount of collagen deposition were scored as follows: (−)=nil, (+)=mild, (++)=moderate and (+++)=marked. There were mild chronic inflammatory cells, moderate to marked fibroblast proliferation, no collagen deposition, mild to moderate haemorrhage, mild haemosiderin deposition and a lack of mineralized deposits. At 1 month, the moderate to marked acute inflammatory response seen at the 1 week time point continued. Multinucleated giant cells were abundant. Also, similar to the 1 week time point, there was a mild chronic inflammatory response and moderate to marked fibroblast proliferation. Mild collagen deposition was seen, mild haemorrhage in one animal, with moderate haemosiderin deposition and a lack of mineralization. At 6 months, the acute inflammatory response remained mild in the ongoing presence of multinucleated giant cells. The chronic inflammatory response lessened and was mild. Fibroblast proliferation and collagen deposition increased at this time point, with a moderate response on average. Haemorrhage remained nil to mild. Haemosiderin deposition was slightly lower than at earlier time points (mild to moderate on average), and mineralization was mild.

We next characterized the host tissue response generated by the biomimetic devices intended for clinical use. Haematoxylin and Eosin (H&E) staining indicated an acute inflammatory response characterized by the presence of polymorphonuclear cells at short times after device implantation (upper panels in Fig. 2). This initial inflammatory reaction decreased with time, accompanied by a progressive fibrotic response characterized by the migration and proliferation of fibroblasts in the short term, with the appearance of fibrous connective tissue becoming denser with time, and neovascularization and infiltration of host cells within the porous scaffold structure. Similar results could be observed with Gomori's Green Trichrome staining (middle panels in Fig. 2), showing an increasing formation of fibrous tissue with time. The deposition of collagen resulting from the FBR was also demonstrated by immunohistochemistry (lower panels in Fig. 2). Connective tissue stained in brown was observed in the short term after device implantation, and a large amount was present in the medium to long term post-implantation of the device. Importantly, the progressive production of collagen associated with the chronic host fibrotic reaction was accompanied by a sustained durability of the efficacy of tumour cell capture for ≤1 year (Fig. S4). This supports the long-term effectiveness of the biomimetic device, with 97.7% of captured tumour cells at 1 month (*n*=4), 94.3% at 6 months (*n*=12) and 93.3% at 12 months (*n*=6). No statistically significant differences in the efficacy of tumour cell capture by the biomimetic device were observed among the three time points (Fig. S4; *P*=0.7876).

### Efficacy of the biomimetic device in a clinically relevant model of recurrent ovarian cancer

**Fig. 3.**
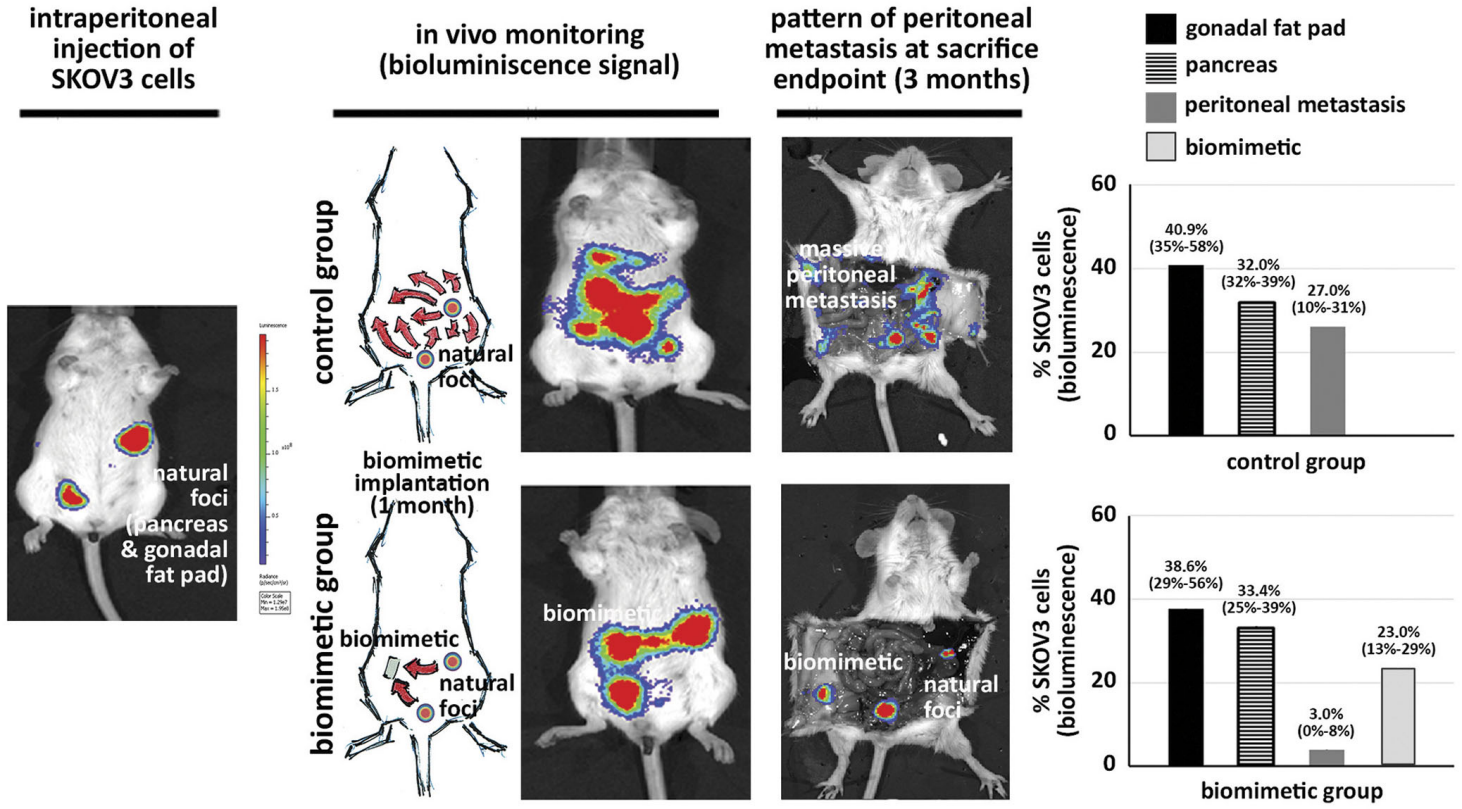
**Efficacy** **of the biomimetic device to capture metastatic SKOV3 cells in a mouse model of recurrent ovarian cancer.** Representative *in vivo* bioluminescence images illustrate the evolution of peritoneal dissemination from existing ovarian metastases generated by peritoneal injection of luciferase-expressing SKOV3 cells resulting in tumour implants in the pancreas and gonadal fat pad as natural sites of metastasis (left panel), in the presence (lower panels; biomimetic group) or absence (upper panels; control group) of the biomimetic devices; normalized photons represented in the colour scale are indicative of the corresponding tumour cells for each animal included in the two study groups. Biomimetic device implantation was performed 1 month after the generation of the orthotopic ovarian tumour, and mice were monitored *in vivo* by bioluminescence for peritoneal dissemination (central panels). The pattern of peritoneal metastasis was analysed by bioluminescence at sacrifice, 3 months after the generation of the recurrent ovarian model (right panels). Histograms show quantification of the distribution of tumour cells in the peritoneal metastasis represented as a percentage of SKOV3 cells by location based on the bioluminescence signal (normalized photons). Black bars indicate the amount of tumour cells in the originated ovarian metastasis in the pancreas, and lined bars correspond to metastasis in the gonadal fat pad, as a natural site of metastasis; grey bars represent the percentage of SKOV3 cells in implants distributed in other peritoneal locations and organs different from natural sites; and the pale grey bar in the biomimetic group corresponds to the tumour cells captured by the devices. Of note, peritoneal metastases are drastically reduced in the biomimetic group compared with the control group, indicating a remodelling of the pattern of peritoneal dissemination in the presence of the biomimetic device in this model of recurrent ovarian cancer (*P*=0.008; Mann-Whitney *U* test).

After characterizing the mode of action of the biomimetic technology and the impact of the FBR, we assessed the efficacy of tumour cell capture in a murine model of advanced ovarian cancer mimicking a recurrent disease setting with sustained release of tumour cells from existing peritoneal metastases. For this, we implanted the biomimetic devices after the formation of tumour implants at the natural sites of metastasis, pancreas and gonadal fat pad (post-injection model), by intraperitoneal injection of luciferase-SKOV3 cells (left panel in Fig. 3). Massive dissemination from these tumour lesions was perceptible *in vivo* at the 1.5 month intermediate time point and evident at the 3 month endpoint (central panels in Fig. 3). Upon sacrifice, these animals presented a massive peritoneal carcinomatosis in addition to the original sites of the pancreas and gonadal fat pad (control group; right panel in Fig. 3). In contrast, in the presence of the biomimetic devices this pattern was completely remodelled, with three foci corresponding to the two natural sites of metastasis and to the device containing those cells released from the existing metastases (biomimetic group; right panel in Fig. 3). Quantification of the bioluminescence signal showed that the amount of tumour cells in new peritoneal metastasis was drastically reduced to residual disease in the presence of the biomimetic device (*P*=0.008; histograms in Fig. 3). The devices were able to capture cancer cells in a relevant model mimicking sustained and continuous dissemination of tumour cells in the peritoneal cavity from existing metastasis, mitigating the pattern of massive peritoneal metastasis observed in the control group. Of note, no differences were observed in the total bioluminescence signal (Fig. S5), also indicating that capture of tumour cells by the device does not promote tumour proliferation and does not affect overall tumour burden but does influence the pattern of metastasis. We also demonstrated that the biomimetic device did not contribute to or promote any advantageous proliferative effect on the captured tumour cells, in an *in vivo* model of subcutaneous tumour growth (Fig. S6). Consistently, we also demonstrated that once tumour cells disseminating in the peritoneal cavity had been captured by the device, no dissemination or release of these captured tumour cells occurred from the device (Fig. S7). The ability of the biomimetic technology permanently to retain the tumour cells that have been captured by the device precludes any associated risk of promoting new peritoneal metastasis upon tumour cell capture.

### *In vivo* evaluation of the impact of biomimetic devices on survival outcomes

**Fig. 4.**
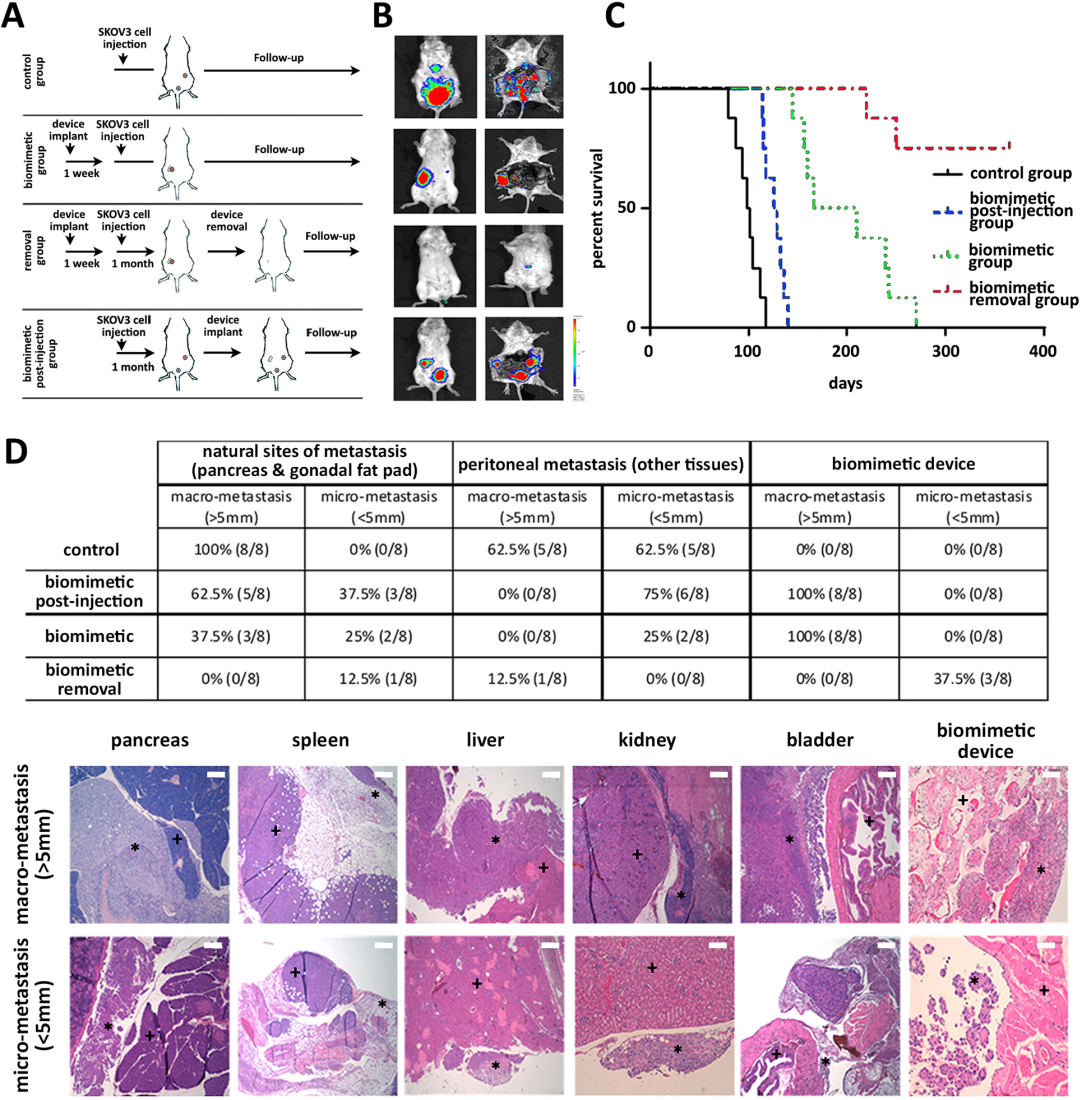
**Design and monitoring of biomimetic survival preclinical trial.** (A) Schematic design of the four-arm biomimetic preclinical study. (B) Capture of tumour cells by the biomimetic device was monitored *in vivo* and at the time of sacrifice. Representative bioluminescence images are shown at the 3-month follow-up (left panels) and at the endpoint (right panels) for each group included in the study; normalized photons represented in the colour scale are indicative of the corresponding tumour cells for each animal included in the four study groups. (C) Kaplan­–Meier survival curves for the different groups included in the study. (D) Histological examination of peritoneal metastasis. Upper table summarizes the percentage of the corresponding micro-metastasis (<5 mm) and macro-metastasis (>5 mm) at the natural sites of metastasis (pancreas and gonadal fat pad), at other peritoneal tissues and within the biomimetic devices, for the different groups included in the study. Lower representative images of a macro- and micro-metastasis are shown for each of these peritoneal tissues; the corresponding peritoneal tissue (+) and the tumour lesion (*) are indicated (scale bars: 500 µm).

The biomimetic device has been designed for use in a clinical setting in conjunction with current therapeutic strategies, including surgery and chemotherapy, with its clinical success being based on its ability to transform a systemic disease into a localized disease. We assessed the impact on survival outcomes in a preclinical study that simulated its intended clinical use (diagram in Fig. 4A). In the control arm, 2.5 million luciferase-SKOV3 cells were injected intraperitoneally and followed up to determine the survival times for the natural pattern of massive peritoneal carcinomatosis (control group, Fig. 4A; *n*=8). In the biomimetic group, the devices were surgically implanted in the inner peritoneal wall of mice, and, 1 week after surgery, tumour cells were injected intraperitoneally (biomimetic group, Fig. 4A; *n*=8). This group represented survival benefits attributable to the focalization of the peritoneal disease. In a second scenario intended to mimic recurrent disease, the devices were surgically implanted 1 month after intraperitoneal injection of tumoural cells (biomimetic post-injection group, Fig. 4A; *n*=8). In a third scenario, the devices were implanted, and tumour cells were injected intraperitoneally 1 week after surgical implantation. One month after injection of tumour cells, the devices were surgically removed (biomimetic removal group, Fig. 4A; *n*=8). This group represents survival benefits attributable to surgical removal, the intended clinical use of the device.

Once the study endpoint was reached, defined as a decrease in the performance status of the mice, according to the Directive 2010/63/EU guideline, the animal was sacrificed. Bioluminesce images illustrate the focalization of peritoneal disease within the device in the biomimetic group, and within the device in addition to the pancreas and gonadal fat pad in the biomimetic post-injection group, in comparison to massive peritoneal carcinomatosis in the control group (Fig. 4B). Finally, removal of the biomimetic device upon capture of tumour cells resulted in residual disease in the biomimetic removal group (Fig. 4B). Quantification of peritoneal carcinomatosis showed no differences in tumour burden in the control group compared with the biomimetic and biomimetic post-injection groups (Fig. S8), but demonstrated the ability of the devices to remodel the pattern of peritoneal metastasis (Fig. 4B). Also, device removal resulted in a significant reduction in tumour burden in the biomimetic removal group (*P*<0.0001; Fig. S8).

The impact of tumour cell capture and the consequent focalization of peritoneal carcinomatosis on survival outcome is illustrated in the Kaplan–Meier survival curves (Fig. 4C). Mice included in the control group reached the endpoint at 99 days on average after initiation of the study, whereas mice included in the biomimetic group presented a significant accumulative survival of 199 days on average (*P*<0.0001; green line in Fig. 4C). Likewise, focalization of peritoneal carcinomatosis within the device in the presence of existing metastasis (biomimetic post-injection group) also demonstrated an improved survival rate over the control group, reaching the endpoint at 126 days on average (*P*=0.0005; blue line in Fig. 4C). Finally, surgical removal of the device upon tumour cell capture presented a significant accumulative survival, with six out of eight animals included in this biomimetic removal group reaching the endpoint at 1 year (*P*<0.0001; red line in Fig. 4C).

Histological analysis of peritoneal tissues confirmed that the presence of the biomimetic devices resulted in a significant reduction of the massive peritoneal carcinomatosis shown in mice in the control group, with macro-metastasis in the pancreas and gonadal fat pad as the natural sites of metastasis in all the animals included in this group, and both macro- and micro-metastasis in other peritoneal tissues in five out of eight animals (Fig. 4D). A reduction in the percentage of macro-metastasis in the pancreas and gonadal fat pad was found in the biomimetic post-injection group and an even more pronounced reduction in the biomimetic group (Fig. 4D). No macro-metastases were found in any other peritoneal tissues in those groups, with a progressive reduction in micro-metastasis from the biomimetic post-injection to the biomimetic group (Fig. 4D). Finally, in the biomimetic removal group, only one animal presented with a macro-metastasis in the liver, and another animal presented with a micro-metastasis in the pancreas (Fig. 4D).

All these findings demonstrate a statistically significant benefit in mean survival outcomes resulting from use of the biomimetic devices in three different clinical scenarios. This is especially relevant in the biomimetic removal group (>3 times improved survival in the framework of a 1 year preclinical study), as representative of the planned use of the device in clinical practice.

## DISCUSSION

Metastatic tumour cell growth and survival in distant organs is facilitated by the formation of a pre-metastatic niche that is basically composed of haematopoietic cells, stromal cells and ECM. The creation and evolution of these niches is supported by existing tumour lesions through complex systemic communication involving extracellular vesicles, and results in an improved efficiency of the homing of circulating tumour cells and micro-metastasis formation (Mariscal et al., 2019). Likewise, biomaterial scaffolds systemically influence metastatic progression through manipulation of the tumour microenvironment (Aguado et al., 2018). In this work, we integrated the concept of creating an artificial niche, which might remodel the pattern of tumour dissemination and transform a disseminated disease into a localized disease (de la Fuente et al., 2015), with therapeutic strategies targeting the tumour microenvironment to modulate cancer cell interactions with the ECM (Hansen et al., 2016). This resulted in the design and development of a ‘biomimetic’ ECM to be translated into clinical practice, composed of a nonresorbable three-dimensional scaffold with a type I collagen coating. Collagen is the most abundant protein of the ECM, and type I collagen, in particular, is a well-established cell adhesion molecule with a long history of use in regenerative medicine applications.

The host response to an implanted biomaterial includes the formation of a fibrous capsule consisting of inflammatory immune cells and fibroblasts around the border of the implant. As also demonstrated in this work, tumour cells can home in to an implant owing to the local foreign body response alone. The composition of the immune cells in the foreign body response might differ depending on the host, modulating the formation of a pre-metastatic niche at an ectopic location (Aguado et al., 2017). In addition, the local fibroblasts that participate in the formation of pre-metastatic niches become cancer supportive through the secretion of growth factors and ECM remodelling proteins (Kalluri and Zeisberg, 2006). The implantation of the biomimetic devices resulted in an initial inflammatory reaction characterized by multinucleated giant and polymorphonuclear cells, progressively transforming into a fibrous connective response characterized by the migration and proliferation of fibroblasts, neovascularization and deposition of collagen. This ability of biomaterials to integrate within a host tissue upon implantation facilitates the formation of a defined microenvironment *in vivo*, thus providing an ectopic site for the recruitment of metastatic tumour cells. In addition to the tumour cell capture action demonstrated by the bare porous scaffold, the FBR associated with the recruitment of immune cells and fibroblasts provided additional capture abilities to the device, as shown by the progressive improvement in efficiency correlating with collagen deposition. The definitive design of the biomimetic technology includes the coating of the scaffold with collagen, resulting in a complete and durable, efficient tumour cell capture ability of the biomimetic technology from the moment of implantation in advanced preclinical models of the peritoneal dissemination of ovarian cancer.

Initially, we characterized the mode of action of the biomimetic device, including the adhesive capabilities of the nonresorbable scaffold and the ECM coating together with the ancillary effect of the FBR. In addition, we have presented performance and safety studies demonstrating its clinical utility in terms of long-term efficacy of tumour cell capture and survival benefit in different preclinical models of advanced ovarian cancer recurrent disease. These preclinical models are representative of the different clinical settings in advanced ovarian cancer, with residual disease after surgery as the main challenge impacting overall survival. The biomimetic model represents a recurrent disease after an R0 surgery with complete tumour resection, whereas the post-injection model mimics a recurrence after an R1-R2 surgery with microscopic-macroscopic residual tumour after primary debulking surgery. Ovarian cancer, accounting for 4% of all cancers in women and usually diagnosed at advanced stages III-IV [International Federation of Gynecology and Obstetrics (FIGO)], is treated by cytoreductive surgery to eliminate the intra-abdominal tumour burden followed by platinum- and taxane-based chemotherapy (Openshaw et al., 2015). To this end, we provided further evidence for the translational benefit of this technology in this specific clinical setting by evaluating the efficacy of the biomimetic device with or without the presence of standard chemotherapy in advanced cancer. No significant effect of the devices on the effectiveness of the therapy was observed, nor was there any impact of the standard carboplatin-paclitaxel regimen on the efficacy of tumour cell capture by the biomimetic device (Fig. S9). Alternatively, neoadjuvant chemotherapy followed by interval debulking surgery can be performed to reduce tumour volume and improve resectability, but, despite radical surgery and chemotherapy, most patients with ovarian cancer develop recurrence and die owing to progressive disease (Armstrong et al., 2006; Cannistra, 2004). Relapse of the disease usually presents as disseminated implants in the peritoneal cavity originating from residual tumour cells resistant to chemotherapy. The time to relapse after platinum-based therapy determines its sensibility/resistance and defines the next clinical decisions, which may include second rescue surgeries in selected patients with localized disease and/or reprise of a platinum-based regimen or second-line therapies, such as inhibitors of poly(ADP-ribose) polymerase (PARP) 1/2, nuclear proteins that detect DNA damage and promote its repair (Mirza et al., 2016).

The possibility that a biomimetic technology for tumour cell capture is able to modify the pattern of peritoneal dissemination in advanced disease upon debulking surgery would represent an opportunity to control the peritoneal carcinomatosis. Implantation of the biomimetic device at the time of debulking surgery in patients diagnosed with early ovarian cancer with localized disease would prevent further peritoneal dissemination. Nevertheless, an eventual impairment of the efficacy of tumour cell capture by the biomimetic technology owing to the extensive debulking surgeries performed in these advanced ovarian cancer patients and to their associated peritoneal adhesions should be examined in larger animal models (i.e. porcine models) before its translation into clinical use. Likewise, the impact of a complete set of immune components, limited in this work by the use of immunocompromised preclinical models, should be evaluated, although preliminary results indicate that the efficacy of tumour cell capture is maintained in immunocompetent models (Fig. S10). In addition, the presence of the devices and their ability to capture tumour cells preferentially could also serve to identify metastatic disease at the earliest stage for patients at risk of recurrence, enabling the initiation of therapy while the disease burden is low (Azarin et al., 2015). Also, the efficacy of tumour cell capture and the focalization of the peritoneal disease, with the consequent benefit in survival demonstrated in the orthotopic model (Fig. 4), indicates that the biomimetic technology maintains a setting of localized disease. This transformation of a massively disseminated disease into a controlled, localized peritoneal carcinomatosis represents per se the possibility to improve the management of advanced ovarian cancer patients at relapse, as demonstrated in the more aggressive preclinical model with existing metastasis (post-injection model in Figs 3 and 4). Translated into the clinics, this biomimetic device would signify the possibility to control the peritoneal carcinomatosis upon primary surgery and to expand the percentage of patients who are candidates for second rescue surgeries at the time of relapse. The additional removal of tumour cells captured by the devices during this rescue surgery together with any residual tumour foci should have a positive impact on patient outcomes (biomimetic removal group in Fig. 4), with the possibility of the implantation of new devices to trap residual disseminating tumour cells. Preliminary data from the prospective AGO-DESKTOP III provide evidence that surgery for recurrent ovarian cancer seems to be of benefit for selected patients (Pignata et al., 2017).

In conclusion, we described the mode of action of a biomimetic device composed of a biomaterial nonresorbable scaffold coated with an ECM protein. We characterized the foreign body response resulting from the implantation of the device supporting a durable tumour cell capture efficacy. We also demonstrated the performance and safety of the biomimetic devices in preclinical models mimicking the clinical setting in advanced ovarian cancer. Finally, we showed the benefit of the biomimetic technology resulting from the focalization of the peritoneal carcinomatosis after surgical implantation and removal upon tumour cell capture. All these results guarantee the design and implementation of a clinical trial in advanced ovarian cancer patients.

## MATERIALS AND METHODS

### Cell lines

The luciferase-expressing human SKOV3 ovarian cell line was purchased from Cell Biolabs [SKOV3-Luciferase (AKR-232); RRID:CVCL_M089] and maintained in McCoy's 5A complete medium (10% fetal bovine serum, 1% penicillin/streptomycin). This human cell line has been authenticated using short tandem repeat profiling within the past 3 years. All experiments were performed with mycoplasma-free cells.

### Ovarian cancer models

Female severe combined immunodeficient (SCID) CB-17/lcr-Prkdcscid/scid/Rj mice (Barcelona Biomedical Research Park, Barcelona, Spain) aged 6-8 weeks were used. Mouse models of advanced ovarian cancer were generated by intraperitoneal injection of 100 μl PBS containing 1 million SKOV3-luciferase cells in the peritoneal cavity; alternatively, for survival studies, 2.5 million cells were injected intraperitoneally.

Biomimetic ECM devices are composed of a polycarbonate polyurethane scaffold with a type I collagen coating (Fig. S1; MTrap, Inc., Huntington, NY, USA). Collagen is the most abundant protein of the ECM, and type I collagen, in particular, is a well-established cell adhesion molecule with a long history of use in regenerative medicine applications. The polycarbonate polyurethane scaffold is a nonresorbable, reticulated, three-dimensional scaffold that has been shown in preclinical studies to serve as an effective bioengineered bone marrow niche (Vaiselbuh et al., 2010). Additionally, clinical studies have demonstrated safe and effective use of the polyurethane scaffold for applications in soft tissue repair (Encalada-Diaz et al., 2011).

Biomimetic devices were surgically implanted through a midline ventral incision. The dimensions of the preclinical devices (6 mm×3 mm×2 mm) were designed based on an eventual surgical laparoscopic implantation of scaled clinical biomimetic devices in a future clinical trial. The maximal concentration of the collagen coating was determined by assessment of device permeability and scanning electron microscopy to achieve acceptable collagen coverage throughout the porous scaffold structure without compromising permeability, supporting cellular infiltration and proliferation (data not shown). The device was fixed to the mesothelium opposite to the pancreas on the inner wall of the peritoneum using a surgical glue (glubran^®^2; GEM S.R.L. ref. G-ND-2, Italy). The wound was closed using Ethicon VICRIL^®^ (Johnson & Johnson international, Diegem, Belgium) suture. Isoflurane (IsoFlo^®^; Esteve, Maidenhead, UK) at a concentration of 2% isoflurane in air was used for general anaesthesia. Buprenodale (Dechra, Dales Pharmaceuticals, Skipton, UK) was used for postoperative analgesia by subcutaneous injection at 1 mg/kg.

Mice were housed and maintained in specific pathogen-free conditions, and used in accordance with institutional guidelines, approved by the Use Committee for Animal Care from the University of Santiago de Compostela. Mice were sacrificed based on the Directive 2010/63/EU on the protection of animals used for scientific purposes. For removal, biomimetic devices were surgically resected by laparotomy using an electric surgical knife. The wound was closed using Ethicon VICRIL^®^ suture.

### Evaluation of peritoneal metastasis

An IVIS system (Xenogen, Caliper Life Sciences, PerkinElmer, Waltham, MA, USA) coupled to Living Imaging software v.4.2 (Xenogen) was used to detect the pattern of peritoneal metastasis by bioluminescence imaging. Luciferin (Firefly Luciferin; Caliper Life Science, Hopkinton, MA, USA) was used as the substrate for the luciferase-expressing tumour cells and injected intraperitoneally at a concentration of 150 mg/kg in PBS. The bioluminescence signal was used to quantify the number of tumour cells as normalized photons within each study, and a corresponding colour scale was used to represent them in the *in vivo* images.

### Histological analysis

Tissue samples and removed devices were immersion fixed in 10% buffered formalin for 24 h, dehydrated in an ethanol series, embedded in paraffin, sectioned (3 μm thick) and mounted on microslides. After drying, dewaxing and rehydratation, tissues were stained with H&E and examined using a LEICA DMD108 digital microscope (LEICA Microsystems, Wetzlar, Germany). The histological analysis of the FBR at the different time points was performed by two independent pathologists, and a minimum of three random fields were evaluated using a semi-quantitative score that considered the amount of inflammatory cells (acute inflammatory cells, chronic inflammatory cells and multinucleated macrophages), the presence of erythrocytes, haemosiderin deposition, mineralized deposits and the amount of collagen deposition and scored these as follows: (−)=nil, (+)=mild, (++)=moderate and (+++)=marked. Alternatively, the histological analysis of micro- and macro-metastases in the survival study was performed in at least three H&E sections processed from the paraffin block at intervals of 30 µm and examined by two independent pathologists. Tissues positive for the presence of metastasis were evaluated further to determine the size of each metastatic nodule measured with a glass millimetre ruler and classified as micro-metastases (measuring ≤5.0 mm) or macro-metastases (measuring >5.0 mm).

### Gomori's Green Trichrome staining

Formalin-fixed, paraffin-embedded tissue blocks sectioned at 3 μm thickness were dried for 15 min at 65°C before the staining procedure with the Artisan™ Link Special Staining System (Agilent Technologies-DAKO, Santa Clara, CA, USA).

### Immunohistochemical staining

Formalin-fixed, paraffin-embedded tissue blocks were sectioned at a thickness of 3 μm and dried for 1 h at 65°C before the pretreatment procedure of deparaffinization, rehydration and epitope retrieval in the Pre-Treatment Module, PT-LINK (Agilent Technologies-DAKO) at 95°C for 20 min in 50× Tris/EDTA buffer, pH 9, for anti-LYVE1 antibody, and at 95°C for 20 min in 50× citrate buffer, pH 6 for anti-collagen type I antibody (both 1:100 dilution, polyclonal; AB14917 and AB21286, Abcam, Cambridge, UK). Before staining the sections, endogenous peroxidase was blocked. After incubation with the primary antibody, the reaction was visualized with the EnVision™ FLEX Detection Kit (Agilent Technologies-DAKO) for collagen I and with Biotin-SP-AffiniPure Goat Anti-Rabbit IgG (1:200 dilution; 111-065-144, Jackson ImmunoResearch, Westgrove, PA, USA) and streptavidin (1:400 dilution; P0397, Agilent Technologies-DAKO) for collagen type I. Diaminobenzidine chromogen was used as a substrate. Sections were counterstained with Haematoxylin.

### Statistical analysis

The normalized bioluminescence/fluorescence signal at the area of the biomimetic device, compared with that of the rest of the peritoneal tissues, was used to evaluate the efficacy. All experiments were repeated at least three times. Student's *t*-test was used to compare the differences between two groups, and analysis of variance was used to compare the differences among multiple groups. Kaplan–Meier survival curves were used to estimate cumulative survival probabilities using IBM SPSS statistics. The overall *P*-value was calculated using a log-rank test. Statistical significance was set at *P*<0.05.

## Supplementary Material

Supplementary information

## References

[DMM043653C1] Aguado B. A., Bushnell G. G., Rao S. S., Jeruss J. S. and Shea L. D. (2017). Engineering the pre-metastatic niche. *Nat. Biomed. Eng.* 1, 0077 10.1038/s41551-017-0077 28989814 PMC5628747

[DMM043653C2] Aguado B. A., Hartfield R. M., Bushnell G. G., Decker J. T., Azarin S. M., Nanavati D., Schipma M. J., Rao S. S., Oakes R. S., Zhang Y. et al. (2018). Biomaterial scaffolds as pre-metastatic niche mimics systemically alter the primary tumor and tumor microenvironment. *Adv. Healthc. Mater.* 7, e1700903 10.1002/adhm.201700903 29521008 PMC6014830

[DMM043653C3] Anderson J. M., Rodriguez A. and Chang D. T. (2008). Foreign body reaction to biomaterials. *Semin. Immunol.* 20, 86-100. 10.1016/j.smim.2007.11.004 18162407 PMC2327202

[DMM043653C4] Armstrong D. K., Bundy B., Wenzel L., Huang H. Q., Baergen R., Lele S., Copeland L. J., Walker J. L. and Burger R. A. (2006). Intraperitoneal cisplatin and paclitaxel in ovarian cancer. *N. Engl. J. Med.* 354, 34-43. 10.1056/NEJMoa052985 16394300

[DMM043653C5] Azarin S. M., Yi J., Gower R. M., Aguado B. A., Sullivan M. E., Goodman A. G., Jiang E. J., Rao S. S., Ren Y., Tucker S. L. et al. (2015). In vivo capture and label-free detection of early metastatic cells. *Nat. Commun.* 6, 8094 10.1038/ncomms9094 26348915 PMC4563812

[DMM043653C6] Cannistra S. A. (2004). Cancer of the ovary. *N. Engl. J. Med.* 351, 2519-2529. 10.1056/NEJMra041842 15590954

[DMM043653C7] Colombo N., Van Gorp T., Parma G., Amant F., Gatta G., Sessa C. and Vergote I. (2006). Ovarian cancer. *Crit. Rev. Oncol. Hematol.* 60, 159-179. 10.1016/j.critrevonc.2006.03.004 17018256

[DMM043653C8] de La Fuente A., Alonso-Alconada L., Costa C., Cueva J., Garcia-Caballero T., Lopez-Lopez R. and Abal M. (2015). M-trap: exosome-based capture of tumor cells as a new technology in peritoneal metastasis. *J. Natl. Cancer Inst.* 107, djv184 10.1093/jnci/djv184 26150590 PMC4836824

[DMM043653C9] Encalada-Diaz I., Cole B. J., Macgillivray J. D., Ruiz-Suarez M., Kercher J. S., Friel N. A. and Valero-Gonzalez F. (2011). Rotator cuff repair augmentation using a novel polycarbonate polyurethane patch: preliminary results at 12 months’ follow-up. *J. Shoulder Elbow Surg.* 20, 788-794. 10.1016/j.jse.2010.08.013 21106404 PMC3872973

[DMM043653C10] Hansen J. M., Coleman R. L. and Sood A. K. (2016). Targeting the tumour microenvironment in ovarian cancer. *Eur. J. Cancer* 56, 131-143. 10.1016/j.ejca.2015.12.016 26849037 PMC4769921

[DMM043653C11] Høye A. M. and Erler J. T. (2016). Structural ECM components in the premetastatic and metastatic niche. *Am. J. Physiol. Cell Physiol.* 310, C955-C967. 10.1152/ajpcell.00326.2015 27053524

[DMM043653C12] Kalluri R. and Zeisberg M. (2006). Fibroblasts in cancer. *Nat. Rev. Cancer* 6, 392-401. 10.1038/nrc1877 16572188

[DMM043653C13] Kenny H. A., Chiang C.-Y., White E. A., Schryver E. M., Habis M., Romero I. L., Ladanyi A., Penicka C. V., George J., Matlin K. et al. (2014). Mesothelial cells promote early ovarian cancer metastasis through fibronectin secretion. *J. Clin. Invest.* 124, 4614-4628. 10.1172/JCI74778 25202979 PMC4191043

[DMM043653C14] Lambert L. A. (2015). Looking up: recent advances in understanding and treating peritoneal carcinomatosis. *CA Cancer J. Clin.* 65, 284-298. 10.3322/caac.21277 25940594

[DMM043653C15] Lemoine L., Sugarbaker P. and Van Der Speeten K. (2016). Pathophysiology of colorectal peritoneal carcinomatosis: role of the peritoneum. *World J. Gastroenterol.* 22, 7692-7707. 10.3748/wjg.v22.i34.7692 27678351 PMC5016368

[DMM043653C16] Lengyel E. (2010). Ovarian cancer development and metastasis. *Am. J. Pathol.* 177, 1053-1064. 10.2353/ajpath.2010.100105 20651229 PMC2928939

[DMM043653C17] Lu P., Weaver V. M. and Werb Z. (2012). The extracellular matrix: a dynamic niche in cancer progression. *J. Cell Biol.* 196, 395-406. 10.1083/jcb.201102147 22351925 PMC3283993

[DMM043653C18] Mariscal J., Fernandez-Puente P., Calamia V., Abalo A., Santacana M., Matias-Guiu X., Lopez-Lopez R., Gil-Moreno A., Alonso-Alconada L. and Abal M. (2019). Proteomic characterization of epithelial-like extracellular vesicles in advanced endometrial cancer. *J. Proteome Res.* 18, 1043-1053. 10.1021/acs.jproteome.8b00750 30585730

[DMM043653C19] Mikuła-Pietrasik J., Uruski P., Tykarski A. K. and Książek K. (2018). The peritoneal “soil” for a cancerous “seed”: a comprehensive review of the pathogenesis of intraperitoneal cancer metastases. *Cell. Mol. Life Sci.* 75, 509-525. 10.1007/s00018-017-2663-1 28956065 PMC5765197

[DMM043653C20] Mirza M. R., Monk B. J., Herrstedt J., Oza A. M., Mahner S., Redondo A., Fabbro M., Ledermann J. A., Lorusso D., Vergote I. et al. (2016). Niraparib maintenance therapy in platinum-sensitive, recurrent ovarian cancer. *N. Engl. J. Med.* 375, 2154-2164. 10.1056/NEJMoa1611310 27717299

[DMM043653C21] Openshaw M. R., Fotopoulou C., Blagden S. and Gabra H. (2015). The next steps in improving the outcomes of advanced ovarian cancer. *Womens Health* 11, 355-367. 10.2217/WHE.15.6 26102473

[DMM043653C22] Peinado H., Lavotshkin S. and Lyden D. (2011). The secreted factors responsible for pre-metastatic niche formation: old sayings and new thoughts. *Semin. Cancer Biol.* 21, 139-146. 10.1016/j.semcancer.2011.01.002 21251983

[DMM043653C23] Peinado H., Zhang H., Matei I. R., Costa-Silva B., Hoshino A., Rodrigues G., Psaila B., Kaplan R. N., Bromberg J. F., Kang Y. et al. (2017). Pre-metastatic niches: organ-specific homes for metastases. *Nat. Rev. Cancer* 17, 302-317. 10.1038/nrc.2017.6 28303905

[DMM043653C24] Pignata S., C Cecere S., Du Bois A., Harter P. and Heitz F. (2017). Treatment of recurrent ovarian cancer. *Ann. Oncol.* 28 suppl. 8, viii51-viii56. 10.1093/annonc/mdx441 29232464

[DMM043653C25] Pradeep S., Kim S. W., Wu S. Y., Nishimura M., Chaluvally-Raghavan P., Miyake T., Pecot C. V., Kim S.-J., Choi H. J., Bischoff F. Z. et al. (2014). Hematogenous metastasis of ovarian cancer: rethinking mode of spread. *Cancer Cell* 26, 77-91. 10.1016/j.ccr.2014.05.002 25026212 PMC4100212

[DMM043653C26] Siegel R. L., Miller K. D. and Jemal A. (2018). Cancer statistics, 2018. *CA Cancer J. Clin.* 68, 7-30. 10.3322/caac.21442 29313949

[DMM043653C27] Sleeman J. P. (2012). The metastatic niche and stromal progression. *Cancer Metastasis Rev.* 31, 429-440. 10.1007/s10555-012-9373-9 22699312 PMC3470821

[DMM043653C28] Sodek K. L., Murphy K. J., Brown T. J. and Ringuette M. J. (2012). Cell-cell and cell-matrix dynamics in intraperitoneal cancer metastasis. *Cancer Metastasis Rev.* 31, 397-414. 10.1007/s10555-012-9351-2 22527451 PMC3350631

[DMM043653C29] Vaiselbuh S. R., Edelman M., Lipton J. M. and Liu J. M. (2010). Ectopic human mesenchymal stem cell-coated scaffolds in NOD/SCID mice: an in vivo model of the leukemia niche. *Tissue Eng. C Methods* 16, 1523-1531. 10.1089/ten.tec.2010.0179 20586611

[DMM043653C30] Worzfeld T., Pogge Von Strandmann E., Huber M., Adhikary T., Wagner U., Reinartz S. and Müller R. (2017). The unique molecular and cellular microenvironment of ovarian cancer. *Front. Oncol.* 7, 24 10.3389/fonc.2017.00024 28275576 PMC5319992

