# Supplementary material for: Biomimetic device and foreign body reaction cooperate for efficient tumour cell capture in murine advanced ovarian cancer
Source: Dis Model Mech. 2020 Jun 17;13(6):dmm043653. doi: 10.1242/dmm.043653 (PMC7328160; doi:10.1242/dmm.043653)
Supplement: Supplementary information [file dmm-13-043653-s1.pdf]

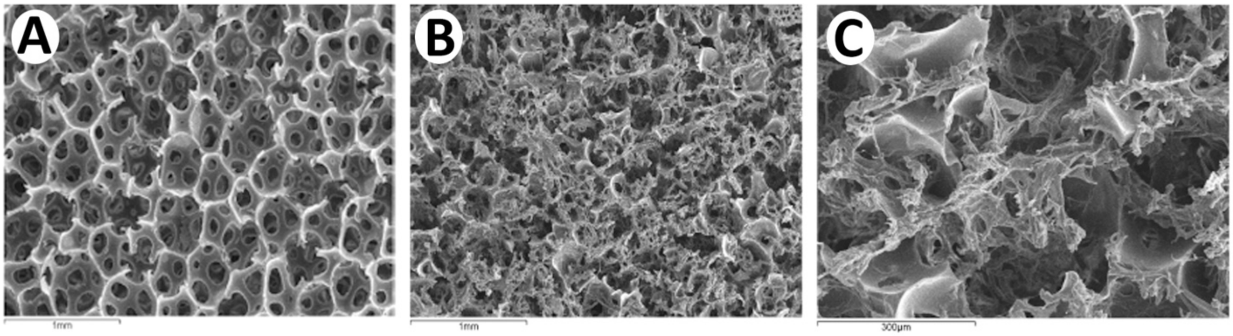

Figure S1

Electron microscopy images of then biomimetic devices showing the polyurethane scaffold and the collagen fibers dispersed within the polyurethane scaffold. A) polycarbonate polyurethane scaffold showing the open cell, reticulated, intercommunicating network present throughout the scaffold structure. B) lyophilized collagen network distributed within the polyurethane scaffold. C) magnification of the lyophilized collagen network.

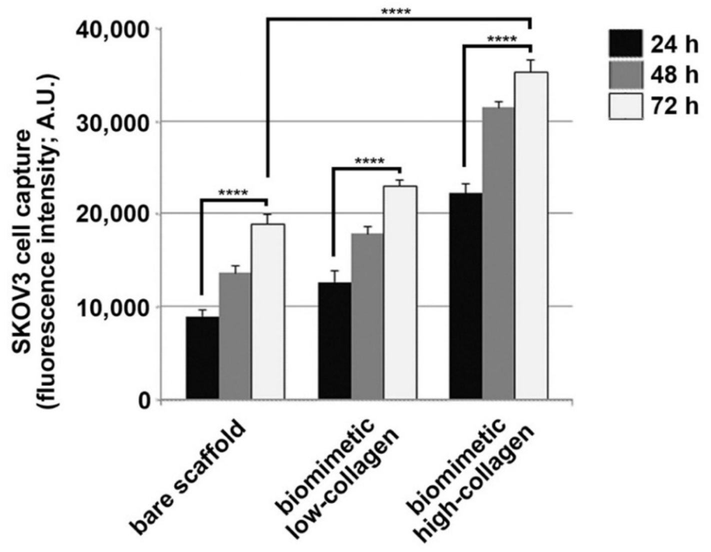

Figure S2

In vitro biomimetic tumor cell capture efficacy was assessed in an orbital adhesion assay that mimics peritoneal dissemination in ovarian cancer (de la Fuente et al., J Natl Cancer Inst. 2015;107(9)). Biomimetic devices, including the bare scaffold without collagen coating and devices with increasing (low and high) concentrations of collagen (25 and 250 mg per device, respectively), were immobilized in pre-treated p100 cell culture plates (one device per plate) and one million of SKOV3 cells, labelled with the fluorescent marker calcein (4 µM/l calcein acetoxymethyl ester (Invitrogen, Paisley, UK), according to manufacturer´s protocol), were added to each plate before placed on an orbital shaker at 90rpm and incubated for 24, 48 and 72 hours at 37ºC in 5% CO2. After incubation, biomimetic devices were recovered from the plates and the amount of cells captured by the bare scaffold and by scaffolds coated with increasing amounts of collagen was evaluated. Quantification of captured fluorescent-labelled SKOV3 cells was performed using a fluorimeter. Fluorescence intensity of calcein is recorded at 485nm wavelength and expressed in arbitrary units. The amount of tumor cells, expressed as mean average ± standard deviation, captured by Biomimetic bare scaffold (Empty Group), and bare scaffold coated with collagen at low (Biomimetic Low Collagen Concentration Group) and high (Biomimetic High Collagen Concentration Group) collagen concentrations at 24 (black bars), 48 (dark grey bars) and 72 (light grey bars) hours of incubation in the adhesion orbital assay, confirmed the principal adhesive abilities of the scaffold and the ancillary effect of the collagen coating ($p<0.0001$), in a collagen concentration and time-dependent manner ($p<0.0001$).

Disease Models & Mechanisms • Supplementary information

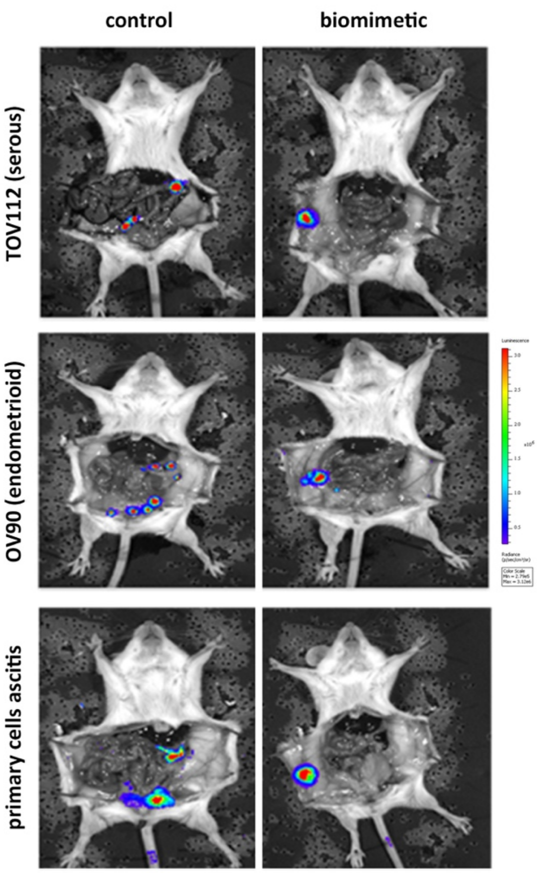

Figure S3

The efficacy of the biomimetic devices to capture different clinically relevant ovarian cancer cell types was evaluated in the murine model of ovarian cancer peritoneal dissemination at one week post-implantation, complementing the results observed with the SKOV3 adenocarcinoma cell line. Two different cell lines were evaluated: TOV112 (serous origin) and OV90 (endometroid origin), as representative cell lines of the most common histology subtypes of ovarian carcinomas diagnosed in the clinical setting. Additionally, primary cancer cells isolated from ascitic fluid of ovarian cancer patients were also studied, representative of patient derived tumors. In this study, one million cells were injected intraperitoneally. One week later, mice were sacrificed and the pattern of tumor cell dissemination was evaluated by bioluminescence for luciferase-expressing TOV112 and OV90 cells (normalized photons; colour scale represents the corresponding number of tumor cells for each animal included in the different study groups with the two cell lines), and by fluorescence for the primary cancer cells isolated from the ascitic fluid of ovarian cancer patients and labelled with fluorescence marker Did. Representative images demonstrated the universality of the biomimetic technology to capture different clinically relevant ovarian cancer cell types. The biomimetic devices (right panels; n=3 for each ovarian cancer cell type) completely remodelled the pattern of peritoneal dissemination with a focalised disease within the devices, compared to the natural pattern of peritoneal dissemination in pancreas and gonadal fat pad in the control groups for TOV112, OV90 and primary ovarian cancer cells (left panels; n=3 for each

Disease Models & Mechanisms • Supplementary information

ovarian cancer cell type). These results confirmed the ability of the biomimetic devices to capture different ovarian cell types disseminating in the peritoneal cavity.

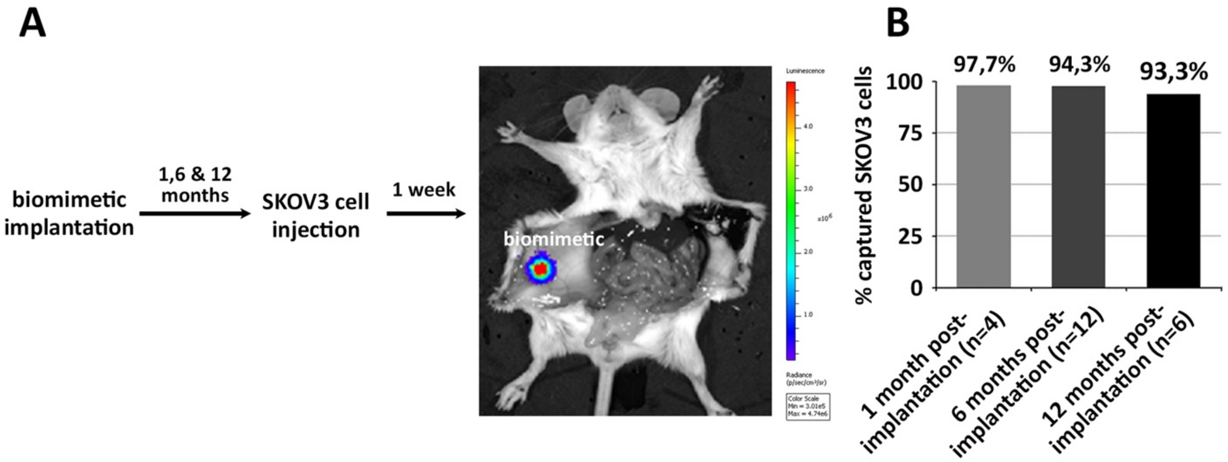

Figure S4

Long term durability of tumor cell capture efficacy of the biomimetic devices. (A) Schematic representation of the study. Biomimetic devices were surgically implanted in the peritoneal wall of female SCID Beige mice aged six-eight weeks, and one million luciferase-expressing SKOV3 cells were injected one (n=4), six (n=12) and twelve (n=6) months later. One week after tumor cell injection, animals were sacrificed and the pattern of tumor cell dissemination was evaluated by bioluminescence, as described in Material and Methods; normalized photons represented in the color scale are indicative of the corresponding tumor cells for each animal included in the three study groups. (B) The percentage of tumor cells captured by the biomimetic device, represented as average bioluminescence signal at the device area, compared to tumor cells in the rest of the peritoneal cavity, for the three groups included in the study, confirmed that the efficacy of biomimetic technology is durable for up to one-year post-implantation, in this in vivo model of metastatic ovarian peritoneal dissemination. A one-way ANOVA analysis at the 95% confidence level using GraphPad Prism 6.0 software, showed no statistically significant difference in tumor cell capture efficacy of the biomimetic devices among the three time-points ($p=0.7876$). Likewise, unpaired t-tests showed no statistical significance among groups, further confirming the durability of the biomimetic technology efficacy for up to one year.

Disease Models & Mechanisms • Supplementary information

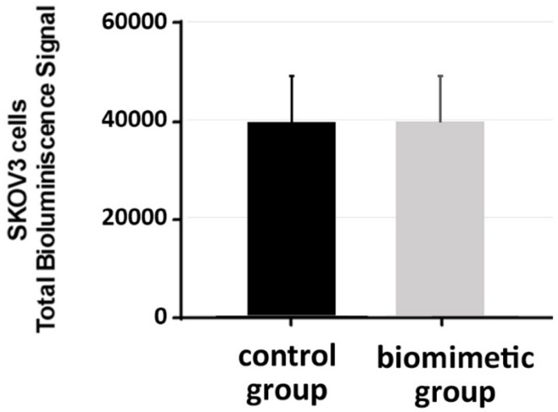

Figure S5

Tumor burden at the time of sacrifice of mice included in the study of the post-injection murine model of recurrent advanced ovarian cancer with sustained release of metastatic cells, evaluated as global bioluminescence signal from tumor cells in the peritoneal metastasis, in addition to the biomimetic devices for the biomimetic group. Normalized photons indicate that no difference was found between the control and the biomimetic groups, further supporting the conclusion that tumor capture efficacy by the biomimetic technology promotes a remodelling of the pattern of metastasis and the focalization of the disease.

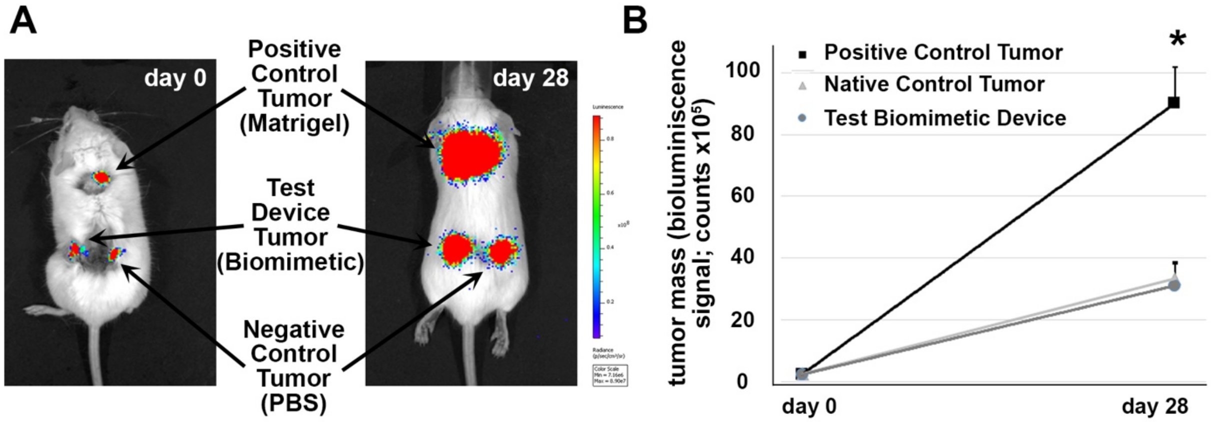

Figure S6

Impact of the implanted devices on the proliferative effect of captured tumor cells. The risk of promotion of tumor growth and proliferation due to use of the biomimetic device was evaluated in a murine model of subcutaneous tumor growth. The proliferation of subcutaneous SKOV3 cell tumors within the biomimetic device was compared to negative and positive controls by quantification of the bioluminescence signal at 4 weeks, as described in M&M. A total of five mice were used for this study; each mouse included three different conditions, each with a tumor load of $2.5x10^6$ tumor cells allowing an optimal duration of the study to obtain statistical significance between groups: luciferase expressing SKOV3 cells were (i) embedded in the biomimetic device and implanted subcutaneously in the mice (Test Device Tumor), (ii) injected subcutaneously in PBS (Negative Control Tumor), and (iii) injected subcutaneously in Matrigel as a favourable condition for the promotion of tumor growth (Positive Control Tumor) in the same mice. Briefly, biomimetic devices seeded with tumor cells were prepared by absorption of 2.5 million SKOV3 cells resuspended in 50 microliters of PBS, within each device. Then, a subcutaneous incision was made in the back (left-lower part) of five mice, one device per mice was implanted, and the wound was closed. Next, 2.5 million SKOV3 cells resuspended in PBS were injected contra-lateral to the biomimetic device, in the back (right-lower part) of each mouse, as negative control; and 2.5 million SKOV3 cells resuspended in Matrigel were injected in the back (upper part) of each mouse, as positive control. (A) Representative bioluminescence images at the initiation (left panel: day 0) and at the end (right panel: day 28) of the study; normalized photons represented in the color scale are indicative of the corresponding tumor cells for each animal included in the study. (B) Quantification of tumor mass growth at initiation of the study (day 0) and at the end of the study (day 28), under the three different conditions. Tumor Cells injected in Matrigel (Positive Control Tumor) showed a statistically significant proliferative advantage at day 28 as compared to the basal environment (Negative Control Tumor; $p<0.0001$), and to the biomimetic site (Test Biomimetic Device; $p<0.0001$). No statistically significant differences were found between the Negative Control Tumor and Test Biomimetic Device conditions ($p=0.5767$), indicative of the absence of proliferative support to the captured tumor cells by the biomimetic device.

Disease Models & Mechanisms • Supplementary information

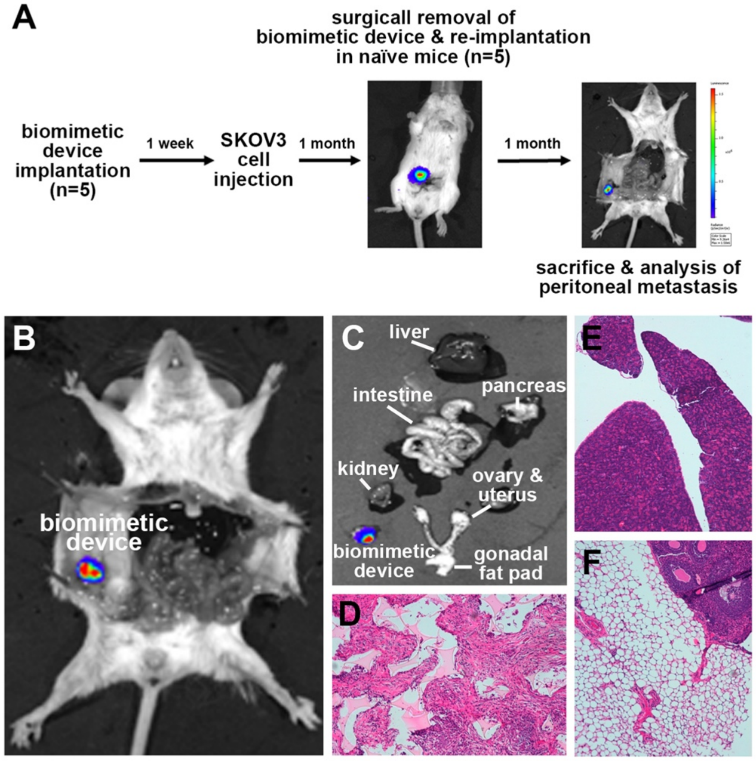

Figure S7

Evaluation of the capacity of the biomimetic device to permanently retain the captured tumor. To evaluate whether any release of tumor cells from the biomimetic device may occur once the tumor cells have been captured by the device, we simulated the implantation of a device with captured cells in a mouse model. (A) Scheme of the evaluation of tumor cell dissemination from the biomimetic device upon tumor cell capture. Five animals were implanted with one biomimetic device per mouse as described in M&M; one week later, one million luciferase expressing SKOV3 cells were injected. After one month, the biomimetic devices with captured tumor cells were removed and re-implanted in five new naïve mice. One month later, mice were sacrificed and the pattern of peritoneal metastasis was analysed by bioluminescence, as described. (B) Representative bioluminescence image of the pattern of peritoneal metastasis of mice re-implanted with the biomimetic device with captured tumor cells. As can be observed, the bioluminescence signal corresponding to the tumor cells remained concentrated within the re-implanted biomimetic device, without any dissemination of tumor cells from the device to the peritoneal cavity, which could be resulting in the appearance of new metastasis (C) Bioluminescence signal of explanted peritoneal tissues confirmed the complete absence of new implants, with tumor cells concentrated within the biomimetic device. In addition, explanted biomimetic devices together with the pancreas and gonadal fat pad as natural sites for peritoneal metastasis, were prepared for histological procedures by preservation in paraffin. Hematoxylin & Eosin (H&E) was used in the histological examination. Microscopic evaluation by two independent pathologists was done to evaluate the absence of tumor lesions at the pancreas and gonadal fat pad, and confirmed the absence of metastatic dissemination from the biomimetic devices with the captured tumor cells, for each animal included in the study. (D-F) H&E image of the (D) biomimetic

Disease Models & Mechanisms • Supplementary information

device with tumor cells infiltrating the pores of the device and the absence of tumor implants at (E) the pancreas and (F) gonadal fat pad. H&E images 10x objective.

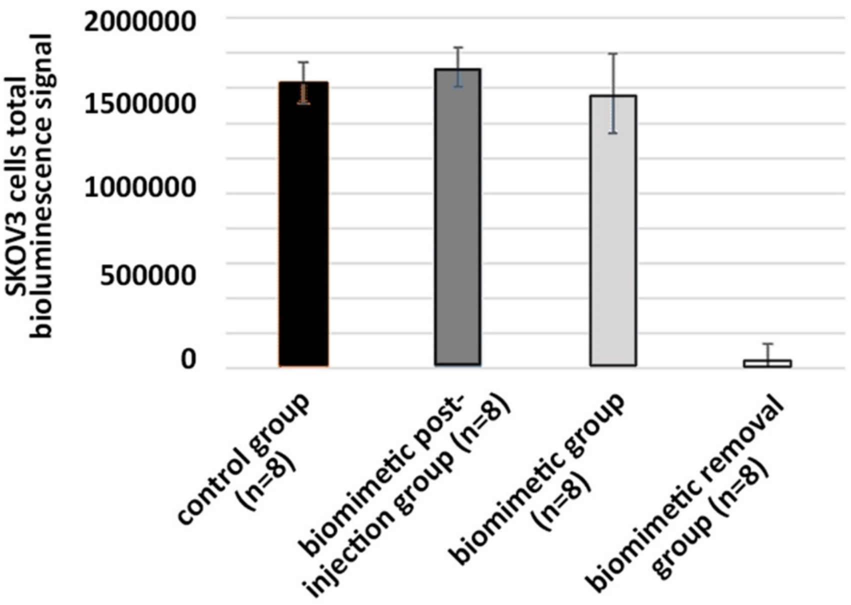

Figure S8

Tumor burden at time of sacrifice of mice included in the survival preclinical study that simulates its intended clinical use evaluated as global bioluminescence signal from tumor cells in the peritoneal metastasis and in the biomimetic devices for the different groups included in the study. Normalized photons indicate that no differences were found between the control, the biomimetic post-injection and the biomimetic groups, further supporting the conclusion that tumor capture efficacy by the biomimetic technology promotes a remodelling of the pattern of metastasis and the focalization of the disease. Finally, bioluminescence signal from mice included in the biomimetic removal group represents residual disease after removal of the devices.

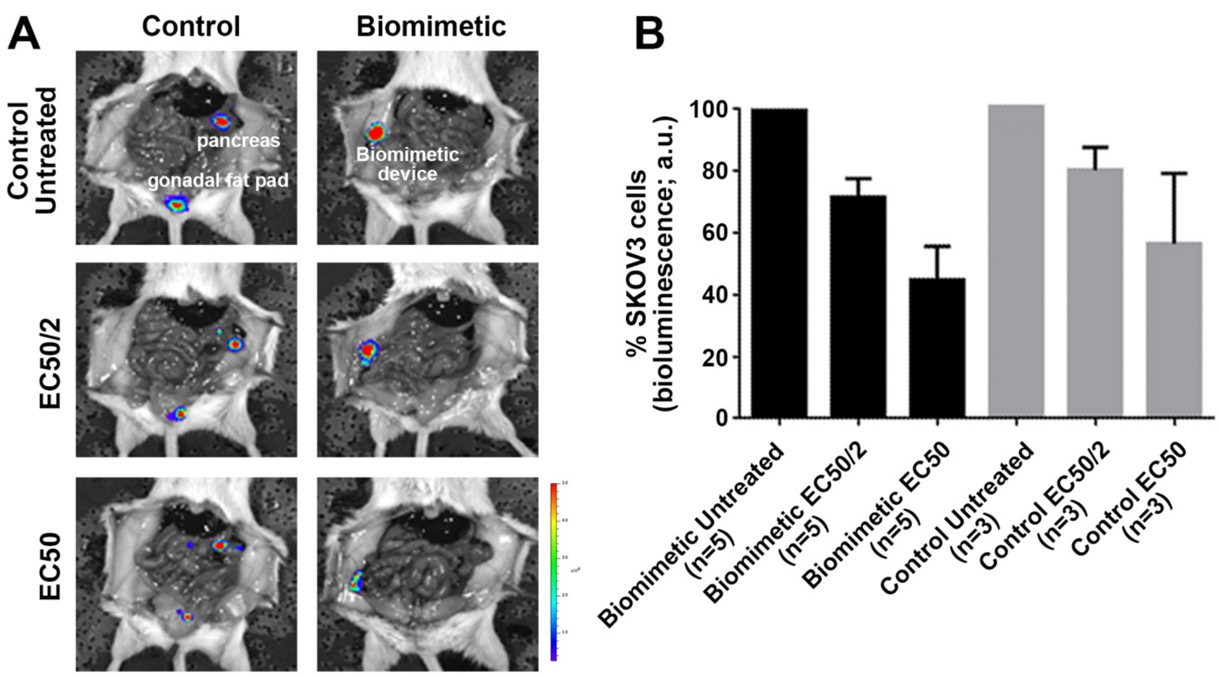

Figure S9

Efficacy of the standard therapy regimen and tumor cell capture efficacy of the biomimetic device in the presence of chemotherapy. The objective of this study was to evaluate the efficacy of the biomimetic device to capture ovarian cancer cells (SKOV3) disseminating in the peritoneal cavity in a mouse model of ovarian cancer (SCID mouse) in the presence of standard chemotherapy administered intraperitoneally (carbotaxol, combination of paclitaxel+carboplatin). This study was also intended to assess whether the presence of the biomimetic tecnology in the peritoneal cavity of mice during treatment affects the efficacy of standard chemotherapy. The pattern of tumor cell dissemination was evaluated by bioluminescence in the presence or absence of the biomimetic device. The Control group was designed to evaluate the efficacy of chemotherapy in the absence of the device, while the Biomimetic group was designed to evaluate the capture efficacy of the device in the presence of EC50 and EC50/2 dosages of standard chemotherapy. EC50 represents the concentration of a drug at which 50% of its maximal effect was observed (Carboplatin TEVA (10mg/ml; lot number: 13E28KB); and Paclitaxel TEVA (6mg/ml; lot number 3620112). EC50 dose for Carboplatin was 10µM, and for Paclitaxel was 4nM; EC50/2 represents half of the EC50 drug concentration. One week after biomimetic device implantation, one million luciferase-expressing SKOV3 ovarian cancer cells were injected intraperitoneally, and 24 hours later chemotherapy was administrated intraperitoneally. The pattern of peritoneal metastasis was

Disease Models & Mechanisms • Supplementary information

analyzed one-week post-injection by bioluminescence, as described in M&M. (A) Representative bioluminescence images of mice injected with one million of luciferase expressing SKOV3 cells in the presence (Biomimetic) and absence (Control) of a biomimetic device implanted one week before; and in the absence of chemotherapy (Control Untreated) or in the presence of chemotherapy (EC50/2 and EC50). As shown, the pattern of peritoneal metastasis in the presence or absence of a biomimetic device was not modified by the concomitant administration of standard chemotherapy. (B) Histogram showing the bioluminescence quantification demonstrated that (i) the capture efficacy of the biomimetic device was not affected in the presence of chemotherapy, and (ii) the efficacy of standard chemotherapy was not altered by the presence of the biomimetic device in the peritoneal cavity.

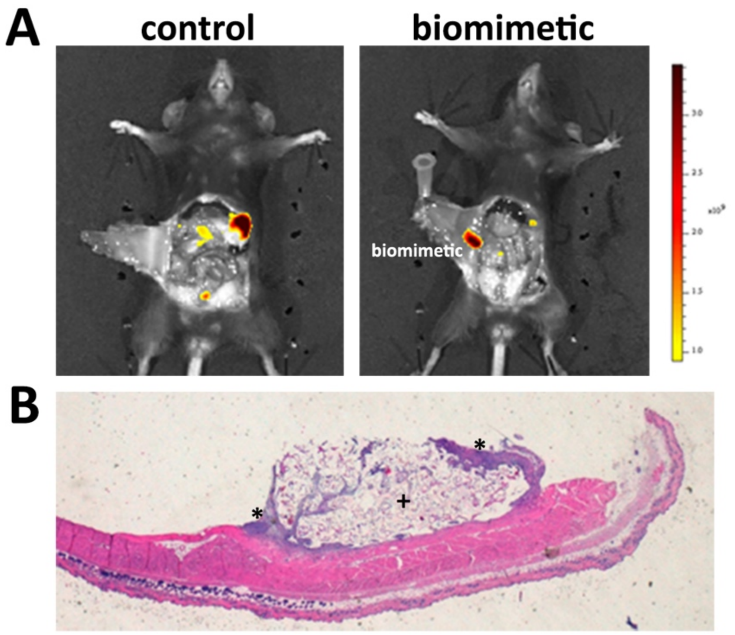

Figure S10

Tumor cell capture efficacy of the biomimetic device in immune-competent mice. To evaluate the impact of the immune system on the tumor cell capture efficacy of the biomimetic technology, immune-competent mice (C57BL/6) were implanted with the biomimetic devices as described in M&M, and one week later injected with $3x10^6$ DiD-fluorescently labelled ID8 ovarian cancer cells. This cell line is originated from mice tumors so the use of immune competent preclinical models is favoured. One week after tumor cell injection, mice were sacrificed and the pattern of peritoneal dissemination evaluated as described in M&M, in the presence (biomimetic group; n=2) and absence (control group; n=2) of the biomimetic device. As shown by these preliminary results, the

Disease Models & Mechanisms • Supplementary information

ability of the biomimetic device to capture tumor cells actively disseminating in the peritoneal cavity and to remodel the pattern of peritoneal metastasis is maintained in the presence of a competent immune system (normalized fluorescence photons represented in the color scale are indicative of the corresponding tumor cells for each animal included in the study groups; panel A). Histology analysis of the explanted biomimetic device (+) confirmed the presence of ID8 tumor cells within the device (*), implanted at the inner wall of the peritoneal cavity (panel B). Adequate studies with humanized preclinical models are ongoing to appropriately characterise the impact of the immune components on the efficacy of the biomimetic technology.

Disease Models & Mechanisms • Supplementary information
